# Musical Training Amplifies Food Cue-Related Interference in Working Memory

**DOI:** 10.3390/bs16050659

**Published:** 2026-04-27

**Authors:** Mingyue Xiao, Yatong Guo, Youjia Song, Yazhi Pang, Pan Shi, Jia Zhao, Yong Liu

**Affiliations:** 1 Key Laboratory of Cognition and Personality, Ministry of Education, Southwest University, Chongqing 400715, China; xiaomy099@163.com (M.X.); gyt1313113@email.swu.edu.cn (Y.G.); youjiaa123456@gmail.com (Y.S.); xiaoshi.pang.20@alumni.ucl.ac.uk (Y.P.); jiazhao@swu.edu.cn (J.Z.); 2 School of Psychology, Southwest University, Chongqing 400715, China; ship1001@swu.edu.cn; 3 Postdoctoral Research Station in Education, Southwest University, Chongqing 400715, China; 4 National Demonstration Center for Experimental Psychology Education, Southwest University, Chongqing 400715, China

**Keywords:** food craving, working memory, musical training, food cues, cognitive interference

## Abstract

**Background**: Musical training has been widely associated with enhanced cognitive abilities, yet its influence on food-related cognitive processing remains largely unexplored. Food craving is known to interfere with working memory (WM), particularly in the presence of highly salient food cues. This study investigated how musical training interacts with food craving to shape WM performance in food-related contexts. **Methods**: Thirty-eight university students with or without musical training completed a food cue 2-back task involving high- and low-calorie food images while electroencephalography (EEG) was recorded. Behavioral performance (reaction time and accuracy), event-related potentials (ERPs), and self-reported food craving were assessed. Mediation and moderation analyses were conducted to examine the role of craving-related dimensions in task performance. **Results**: Participants with musical training showed longer reaction times than non-musically trained participants, while accuracy did not differ between groups. EEG results revealed larger N2 amplitudes in musically trained individuals in response to high-calorie food cues, indicating increased cognitive conflict. Mediation analyses showed that food craving-related intentions and plans indirectly linked musical training to slower task performance, and moderation analyses indicated that this effect was stronger with longer training duration. **Conclusions**: These findings suggest that musical training does not uniformly facilitate working memory in food-related contexts but may heighten sensitivity to motivationally salient food cues, thereby increasing cognitive interference. The study highlights the importance of individual experience and internal states in shaping cognitive responses to food cues and provides new insights into how expertise may influence food-related cognition and decision-making.

## 1. Introduction

Executive functions (EFs) refer to a set of higher-order cognitive processes that regulate an individual’s behavior and thinking when it is necessary to focus attention, suppress impulsivity, flexibly adjust strategies, or process complex information. EFs primarily consist of inhibitory control, working memory (WM), and cognitive flexibility (Diamond, 2013). Among these components, WM plays a central role because it enables individuals to maintain and manipulate information over short periods of time in the service of ongoing goals (A. D. Baddeley, 2013). Classical theoretical models propose that WM consists of three interacting subsystems: the central executive, which coordinates attentional resources; the phonological loop, specialized for verbal information; and the visuospatial sketchpad, dedicated to visual–spatial representations (A. D. Baddeley & Hitch, 1974). WM is commonly assessed using tasks such as the n-back task (Leon-Dominguez et al., 2015), the running memory task (Pollack et al., 1959), and the letter memory task (Cohen et al., 1997). WM is not only involved in information maintenance but also interacts with inhibitory control and cognitive flexibility to achieve successful completion of complex tasks.

Neuroimaging evidence further demonstrates that WM engages a distributed neural network. Specifically, the dorsolateral prefrontal cortex (DLPFC) is implicated in executive control and manipulation, while the posterior parietal cortex (PPC) supports the maintenance of sensory information. Subcortical structures, including the thalamus and basal ganglia, contribute to information gating and coordination (Haque et al., 2021). In the domain of visual working memory, frontoparietal circuits play a central role, with the DLPFC mediating executive control and the PPC underpinning storage and capacity-limited representation (Mayer et al., 2007). Additionally, sensory regions in the occipitotemporal cortex are recruited during encoding and maintenance, with their activation modulated by attentional demands and resource competition between perception and memory processes. WM processing can also be indexed by several event-related potential (ERP) components. In particular, the N2 component has been linked to cognitive control and processing load during WM tasks, reflecting the impact of task difficulty on the allocation of cognitive resources (Kramer et al., 1995). The P2 component is thought to reflect early attentional processing (Cepeda-Freyre et al., 2020), whereas the P3 component has been associated with stimulus evaluation and resource updating during WM performance (Guo et al., 2023). Later positive activity, such as P5, may further reflect post-perceptual processing relevant to task completion (Buss et al., 2014). Beta-band activity is also associated with WM. Increased beta-wave amplitude is indicative of the heightened cognitive demands placed on the brain when maintaining and manipulating information (Scholz et al., 2017). Meanwhile, higher theta amplitude is typically associated with the information encoding and maintenance phases of WM tasks, indicating stronger allocation of cognitive resources and more efficient information processing (Sauseng et al., 2010). WM is influenced by both individual intrinsic factors and external control factors. Among these, diet and musical training have both been shown to exert significant effects on individual WM performance (Blasiman & Was, 2018).

Craving refers to a subjective experience of an intense and urgent desire to consume a substance or perform a behavior, characterized by multidimensional cognitive, emotional, sensory, and physiological components (Hormes & Rozin, 2010; Miele et al., 2023). Food craving, in particular, entails intrusive thoughts, multisensory imagery, and strong motivational impulses toward consumption, often with diminished self-control and anticipation of reinforcement (Taylor, 2019). Tiffany’s (1990) cognitive model posits that craving emerges when nonautomatic processes are activated to inhibit or override automatic behaviors, consuming additional cognitive resources (Tiffany, 1990). Accordingly, craving interferes with cognitive operations requiring controlled processing, such as WM tasks (Baxter & Hinson, 2001). Empirical research shows that high-calorie food cues impair WM performance, especially among individuals with strong craving tendencies (Meule et al., 2012), and that food images can induce craving and interfere with cognitive control (Green et al., 2000). Research has shown that food cues used in the 2-back task can lead to impaired task performance, and that dietary status indirectly influences current food craving after fasting through 2-back task performance (Meule, 2016). Such interference is most pronounced when stimuli are high in caloric value, which robustly engages motivational systems and reduces task efficiency (Gurbuz & Gokce, 2023). The neurobiology of craving involves a distributed cortico-striatal-limbic network, including the ventral striatum, amygdala, anterior cingulate cortex (ACC), and orbitofrontal cortex (OFC), all of which are activated by craving-related cues (Ray & Roche, 2018). Dysregulation of glutamatergic projections from the prefrontal cortex to the nucleus accumbens has been proposed to enhance cue salience and weaken inhibitory control (Kalivas & Volkow, 2005; Koob & Volkow, 2010). Functional alterations in the insula and DLPFC further impair executive regulation during craving, intensifying cognitive conflict and vulnerability to distraction (Courtney et al., 2016). In addition, accumulating evidence suggests that the oxytocinergic system in the central nervous system is also involved in the regulation of eating behavior, food motivation, and food-cue processing. Central oxytocin signaling has been implicated in appetite regulation and food intake, and human studies further show that oxytocin can reduce reward-driven food intake and modulate neural responses to food cues (Kerem & Lawson, 2021; Ott et al., 2013; Plessow et al., 2018; Spetter et al., 2018). Food-craving research has likewise identified oxytocin as a relevant neuroendocrine factor (Huang et al., 2023). These findings highlight the competitive interaction between craving and WM, as both demand overlapping cognitive resources.

Another important factor influencing WM is musical training. Long-term training is associated not only with enhanced music-specific abilities (Habibi et al., 2018) but also with changes in non-musical cognitive functions (Nan, 2017; H. Wang et al., 2015). However, research remains inconclusive regarding the scope and generalizability of these effects. Some studies suggest musicians show superior WM updating abilities (Lu & Greenwald, 2016) and outperform non-musicians in auditory n-back tasks (Ding et al., 2018). Other work argues that the benefits of music training may be domain-specific, mediated by musical WM rather than directly transferred to visuospatial WM (Silas et al., 2022). Age may also be a factor: musically trained children outperform peers in WM tasks, whereas differences between trained and untrained adults are less consistent (Lee et al., 2007). Thus, the influence of musical training on WM appears contingent on age, training type, and task domain. In addition, music-related individual differences may not be fully captured by formal training alone, but may also be reflected in broader musical sophistication, which includes musical skills, perceptual abilities, engagement, and everyday musical behaviors. In the present study, we operationalized musical experience primarily in terms of formal training.

Although food craving and musical training have often been examined separately in relation to cognition, their joint influence on WM may also be theoretically meaningful. Food craving can capture attention and consume cognitive resources that are necessary for successful WM performance, particularly under food-cue exposure (Meule et al., 2012; Tiffany, 1990). At the same time, musical training has been associated with differences in attentional control, executive functioning, and WM-related processing (Pallesen et al., 2010; Zuk et al., 2014). Therefore, musical training may modulate the extent to which food-related cues interfere with WM performance and its neural correlates.

The overarching objective of the present study was to elucidate the nature and extent of the impact of food craving and musical training experience on individuals’ WM. We aimed to determine the influence of food type (high vs. low caloric food) on these variables. We further explored the mechanisms underlying the effects of musical training duration and different subdimensions of food craving on WM, integrating evidence from electroencephalographic (EEG) research to investigate the neural correlates. We formulated the following hypotheses: (1) Higher levels of food craving would be associated with poorer WM performance, as reflected in longer RTs and lower accuracy (ACC) in the 2-back task. (2) High-calorie food cues were expected to elicit greater interference with WM than low-calorie food cues. (3) Individuals with musical training were expected to differ from those without musical training in behavioral performance under food-cue interference. (4) Musically trained individuals were expected to exhibit altered N2 responses during the processing of food-related stimuli, reflecting possible differences in cognitive control demands under interference. Moreover, we explored the relationships among different modalities of data to establish an integrative framework linking neural correlates, behavioral performance, and physiological responses.

## 2. Methods

### 2.1. Participants

In the present study, we recruited two groups of participants: a music training group and a control group. Participants in the music training group had received at least 2 years of formal musical training, whereas those in the control group had no such experience. This classification was intended to operationalize differences in training background; however, it did not constitute a direct assessment of musical expertise or musical sophistication, nor did it control for specific characteristics of musical background, such as instrument type, training intensity, or other detailed aspects of training experience. To achieve sufficient statistical power, the required sample size was calculated before the experiment using G*Power 3.1 (Faul et al., 2007). This study employed a mixed design, which contained a between-group factor (i.e., group) and two within-group factors (i.e., stimuli and trial). A priori power analyses indicated that a minimum of 36 subjects were required to achieve 95% statistical power to detect a moderate effect (f = 0.25) with a significance level of α = 0.05. In this experiment, data from a total of 38 participants who were recruited from a social media platform were collected. All participants provided written consent before completing the experiment. In addition, all participants reported having no history of psychological disorders and normal or corrected-normal vision. The present study was approved by the University Ethics Committee (IRB No. H22020).

### 2.2. Procedure

For standardization, all participants were required to refrain from eating for at least 3 h before the experiment. After entering the laboratory, participants first had their height and weight measured (to calculate BMI), and then completed questionnaires, including basic information, the Food Craving Questionnaire: Trait, and the Visual Analogue Scale. Finally, all participants completed the 2-back task while the EEG data were recorded.

### 2.3. Questionnaires

#### 2.3.1. Visual Analogue Scale (VAS)

Participants rated their hunger and desire to eat on a 100 mm visual analog scale (VAS), ranging from “not at all” to “very much”.

#### 2.3.2. Food Craving Questionnaires-Trait (FCQ-T)

FCQ-T is used to measure trait food craving in all participants. It consists of 37 items (Cepeda-Benito et al., 2000). For each item, participants are required to rate it on a 6-point scale ranging from 1 (never) to 6 (always). The questionnaire comprises nine subscales: (1) Having Intentions and Plans to Consume Food; (2) Anticipation of Positive Reinforcement That May Result From Eating; (3) Anticipation of Relief From Negative States and Feelings as a Result of Eating; (4) Lack of Control Over Eating; (5) Thoughts or Preoccupation With Food; (6) Craving as a Physiological State; (7) Emotions That May Be Experienced Before or During Food Cravings or Eating; (8) Cues That May Trigger Food Cravings; (9) Guilt From Cravings and/or for Giving Into Them. In the present study, the Cronbach’s *α* coefficients for the subscales ranged from 0.835 to 0.912.

### 2.4. Stimuli and 2-Back Task

The food images used in the experiment, categorized as high or low-calorie, were selected from the Chinese food image database (Liu et al., 2022). A total of 60 food images were included, consisting of 30 images each of high-calorie and low-calorie foods.

In this 2-back task, stimuli were presented one by one, and participants were required to respond to every stimulus. Specifically, they were instructed to press the “F” key when the current stimulus was the same as the one presented two trials previously (target trial, Figure S1), and to press the “J” key when the current stimulus was different from the one presented two trials previously (non-target trial). Participants first performed a practice block, which consisted of 20 trials, and received feedback in case of a false response. Only when participants score over 60 points can they enter the test phase. The test phase consisted of blocks with high-calorie food pictures and low-calorie food pictures separately (order of blocks was counterbalanced across subjects). For each block, there were 60 trials, including 30 targets. The order of trials was pseudo-randomized, with the order of target trials equal in both blocks. Each picture was presented for 2000 ms or until a response was made. Between trials, a blank screen was displayed for 500 ms (Figure S1). Thus, each block had a duration of approximately 3 min.

### 2.5. Behavioral Analysis

Descriptive statistics and independent-samples *t*-tests were conducted on the participants’ demographic information, current hunger levels, and scores on each subscale of the FCQ-T. For variables that failed the Levene’s test for equality of variances, the Welch *t*-test results were reported.

For the 2-back task, the primary measures of interest were RT and ACC. When analyzing RT, trials with an RT beyond three standard deviations from the mean or with an incorrect response were excluded from analyses (Li et al., 2020). Specifically, two 2 (group: music trainees and non-music trainees) × 2 (stimuli: high-calorie and low-calorie food) × 2 (trial: target and non-target) mixed-design three-way ANOVAs were conducted on RT for correct responses and ACC, with group serving as a between-subjects factor and stimuli and trial type as within-subjects factors.

Mediation analyses were performed using the PROCESS macro (version 2.16.3) for SPSS 22.0 (Hayes & Preacher, 2013) to examine the indirect effect of group on RT following high-caloric non-target trials via the mediator of Having Intentions and Plans to Consume Food.

Given our focus on the effects of music training and its interaction with food on WM, we examined the Spearman’s rank-order correlations between behavioral, self-report, and ERP results within music trainees. Notably, several moderation effects revealed more nuanced relationships among these variables. Moderation analyses were also conducted using the PROCESS macro for SPSS (Hayes & Preacher, 2013) to examine the indirect effect of Guilt From Cravings and/or for Giving Into Them on task performance via the moderator of music learning duration.

Indirect (mediation and moderation) effects were evaluated with percentile bootstrap confidence intervals based on 5000 bootstrap samples.

### 2.6. EEG Recording Analysis

Brain electrical activities were recorded by a 32-channel amplifier with a sampling frequency of 1000 Hz (ANT Neuro, Berlin, Germany). EEG data were processed with MATLAB R2022a using the EEGLAB toolbox (Delorme & Makeig, 2004). EEG data were filtered with a bandpass finite impulse response (FIR) filter between 0.1 and 30 Hz. The left and the right mastoids were taken as re-reference sites. The continuous EEG data were divided into trials (−200 ms to 1000 ms) according to the different markers of stimuli, a baseline correction (−200 ms to 0 ms) was applied to each trial, and the quality of the EEG data was inspected trial-by-trial. Trials with abnormal artifacts were excluded. Fluctuations (exceeding ± 80 µV) were eliminated (Mölle et al., 2002). The independent components analysis (ICA) method was applied to the EEG data to remove interference factors (e.g., eye movements, electrocardio, etc.) from the data. In the ICA results, components with EOG artifacts and head movement were removed after visual inspections (Liu et al., 2024). The selection of the frontal site (Fz in particular) is justified by its association with cognitive control processes and its anatomical position that does not overlay directly onto the premotor and motor cortices. This strategic placement mitigates the potential confounding influence of button-press-related movements on the integrity of the recorded data. Hence, the site Fz was determined as the optimal location for data analysis, in accordance with previous research (Braboszcz & Delorme, 2011; Jing et al., 2024). Based on the grand-averaged ERP activities and a previous study (Meng et al., 2012), the ERPs and their time windows were as follows: P2, 140–180 ms; N2, 180–240 ms; P3, 240–310 ms; P5, 350–500 ms.

The time–frequency analysis for the EEG data employed a windowed Fourier transform (WFT) with a fixed 250 ms width Hanning window. The WFT yielded a complex time–frequency spectral estimate F (t, f) at each point (t, f) of the time–frequency plane extending from −200 ms to 1000 ms in the time domain, and from 1 Hz to 30 Hz (in steps of 1 Hz) in the frequency domain, for every single trial. A baseline correction was applied at the subject level using the pre-stimulus interval (pre-stimulus −200 to 0 ms) to calculate the change of power according to the formula:

TFD (t, f) = P (t, f) − R (f)

where P (t, f) = |F (t, f)|^2^ is the power spectral density at a given time-frequency point (t, f), and R (f) is the averaged power spectral density of the signal enclosed within the pre-stimulus reference interval (−200 to 0 ms before the onset of the stimulation) for each estimated frequency. In the food two-back task, brain rhythmic activity of the theta (4–8 Hz, 200–400 ms), alpha (8–13 Hz, 400–700 ms), and beta (13–17 Hz, 300–850 ms) were selected in the current analysis.

## 3. Results

### 3.1. Behavioral Results

As shown in Table 1, music trainees had a significantly longer duration of formal music training (M = 6.95 years, SD = 4.37) than non-music trainees (M = 0.00 years, SD = 0.00) [t(36) = 6.183, *p* < 0.001]. For the FCQ-T subscales, music trainees had higher scores on Having Intentions and Plans to Consume Food (M = 9.95, SD = 1.58) compared to non-music trainees (M = 7.37, SD = 1.85) [t(36) = 5.031, *p* < 0.001], and lower scores on Lack of Control Over Eating (M = 14.21, SD = 3.52) compared to non-music trainees (M = 17.21, SD = 3.71) [t(36) = −3.115, *p* = 0.004]. Additionally, music trainees had higher scores on Guilt From Cravings and/or for Giving Into Them (M = 8.58, SD = 2.42) compared to non-music trainees (M = 5.21, SD = 1.61) [t(36) = 6.486, *p* < 0.001]. Other variables (Age, Gender, BMI, Anticipation of Positive Reinforcement, Anticipation of Relief From Negative States, Thoughts or Preoccupation With Food, Craving as a Physiological State, Emotions During Cravings, Cues Triggering Cravings, and Hunger) did not show significant differences between music trainees and non-music trainees (all *p*s > 0.05).

In Table S1, we demonstrated the descriptive statistics of the task performances (RT) of participants. In the analysis of ACC, no significant differences were found across the various conditions (Table S2). To our surprise, in terms of RT, music trainees performed far worse than their no-music-training counterparts in all conditions.

The three-way ANOVA further confirmed the conclusion. The analysis revealed a significant main effect of trial (target) on RT, *F*(1,36) = 12.009, *p* < 0.001, partial *η*^2^ = 0.250, with post-hoc tests indicating that RT was shorter for target trials compared to non-target trials. Additionally, there was a marginally significant main effect of group on RT, *F*(1,36) = 2.943, *p* = 0.095, partial *η*^2^ = 0.076, with post-hoc tests showing that the RT of music trainees was longer than that of non-music trainees. The interaction between trial (target) and group was also significant, *F*(1,36) = 1.698, *p* = 0.045, partial *η*^2^ = 0.201. Simple effect analysis revealed that the RT for non-target trials was significantly longer than that for target trials within music trainees, with a mean difference of 68.684 ms (SE = 17.221, *p* < 0.001), and a 95% confidence interval ranging from 33.759 ms to 103.609 ms. In target trials, a significant difference was observed between music trainees and non-music trainees (*p* = 0.038), with music trainees exhibiting longer RTs compared to non-music trainees.

The main effect of food type was not significant, *F*(1,36) = 0.529, *p* = 0.472, partial *η*^2^ = 0.014. The two-way interaction between food type and group was not significant, *F*(1,36) = 0.068, *p* = 0.796, partial *η*^2^ = 0.002. The two-way interaction between food type and target type was not significant, *F*(1,36) = 2.668, *p* = 0.111, partial *η*^2^ = 0.069. Finally, the three-way interaction among food type, target type, and group was also not significant, *F*(1,36) = 0.035, *p* = 0.853, partial *η*^2^ = 0.001.

### 3.2. ERP Results

**Figure 1:**
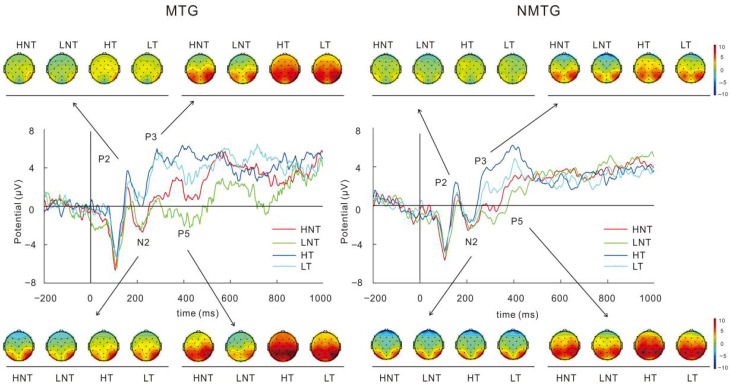
ERPs for N2, P2, P3, and P5 components at Fz. Abbreviations: MTG, music-training group; NMTG, non-music training group; HNT, high caloric non-target trials; HT, high caloric target trials; LNT, low caloric non-target trials; LT, low caloric target trials.

Grand average ERP for N2, P2, P3, and P5 at Fz is shown in Figure 1. Descriptive statistics of those ERP components are shown in Table 2.

#### 3.2.1. N2 Component

It was found that there was a main effect of trial on N2 amplitudes, *F*(1,36) = 8.737, *p* = 0.005, partial *η*^2^ = 0.195, with the post-hoc test indicating that the N2 amplitude of non-target trials was greater than that of target trials; there was also a marginally significant interaction between group and trial, *F*(1,36) = 3.457, *p* = 0.071, partial *η*^2^ = 0.088, and a simple effect analysis showed that among music trainees, the N2 amplitude in non-target trials was significantly greater than that in target trials, *p* = 0.002. There was no significant difference between groups.

#### 3.2.2. P2, P3, and P5 Components

We also conducted analyses on the P2, P3, and P5 components. For the P2 component, the analysis revealed a significant effect of trial type, with target trials exhibiting larger amplitudes than non-target trials: *F*(1,36) = 6.186, *p* = 0.018, partial *η*^2^ = 0.147. For the P3 component, the effect of trial type was highly significant, with target trials showing much larger amplitudes than non-target trials: *F*(1,36) = 23.905, *p* < 0.001, partial *η*^2^ = 0.399. No significant differences were observed for group or stimulus type. For the P5 component, significant effects were found for both the stimuli and trial type dimensions. Regarding stimulus type, high-calorie food stimuli elicited larger P5 amplitudes than low-calorie food stimuli: *F*(1,36) = 4.270, *p* = 0.046, partial *η*^2^ = 0.106. For trial type, target trials again exhibited larger amplitudes than non-target trials: *F*(1,36) = 15.632, *p* < 0.001, partial *η*^2^ = 0.303. No significant differences were observed for the group.

### 3.3. Time–Frequency EEG Results

**Figure 2:**
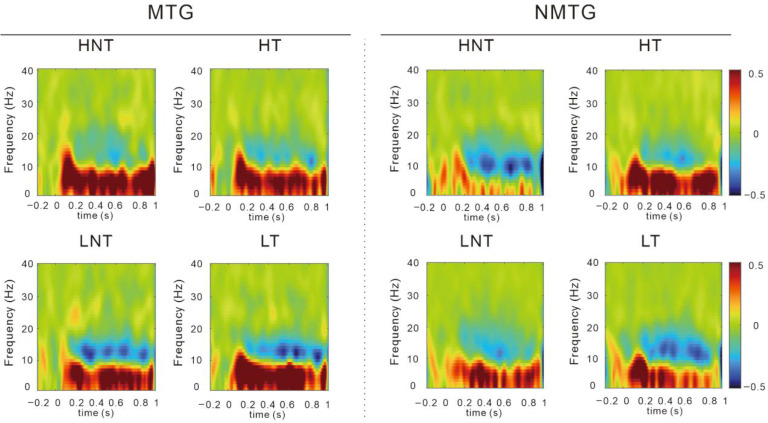
Result of time–frequency analysis. Abbreviations: MTG, music-training group; NMTG, non-music training group; HNT, high caloric non-target trials; HT, high caloric target trials; LNT, low caloric non-target trials; LT, low caloric target trials.

Time–frequency representations are illustrated in Figure 2.

#### 3.3.1. Beta Band Activity

Table 3 shows the descriptive statistics of the beta band. The analysis revealed a significant main effect of trial on average beta amplitudes, *F*(1,36) = 8.451, *p* = 0.006, partial *η*^2^ = 0.190, with post hoc tests indicating that the average beta band power was greater in target trials compared to non-target trials. Additionally, a significant interaction was observed between group and trial, *F*(1,36) = 5.178, *p* = 0.029, partial *η*^2^ = 0.126. Simple effect analysis showed that among non-music trainees, the average beta amplitude in target trials was significantly greater than that in non-target trials (*p* = 0.001).

#### 3.3.2. Other Frequency Bands

Descriptive statistics for theta-band activity are presented in Table 3. The analysis revealed a marginal main effect of trial type on mean theta power, *F*(1,36) = 3.116, *p* = 0.086, partial *η*^2^ = 0.080), indicating a tendency toward greater theta power during target compared to non-target trials. No other main effects or interactions reached statistical significance. No significant main effects or interactions were observed in the alpha band.

### 3.4. Between-Group Mediation Effect

Using Model 4 in PROCESS 4.0, 5000 Bootstrap samples were repeatedly drawn, with group as the independent variable, Having Intentions and Plans to Consume Food as the mediator, and RT following high-caloric non-target trials as the dependent variable, to conduct a moderated mediation analysis.

**Figure 3:**
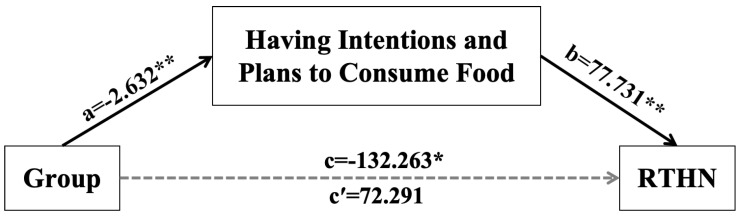
The Mediating Role of Intentions and Plans to Consume Food. Mediation model of an indirect effect of group (music trainees/non-music trainees, independent variable) on RT after high-caloric non-target (outcome variable) via Having Intentions and Plans to Consume Food (mediator). Solid lines indicate significant paths, whereas dotted line indicate non-significant direct effects. The dotted line also indicates that there was an indirect effect of group on RT after high-caloric non-target (bootstrap estimate −0.105, 95%CI [−0.764, −0.354]) in the absence of a total (c) or direct (c′) effect. Note. * indicate *p* < 0.05; ** indicate *p* < 0.01.

There was an indirect effect of group on task performance via food craving (Having Intentions and Plans to Consume Food, as shown in Figure 3). That is, being a musician was indirectly related to RT following high-caloric non-target trials, compared to the control group. This effect, however, was not directly observable but mediated by music trainees’ stronger intentions and plans of food consumption relative to those of non-music trainees.

### 3.5. Relationships Among Self-Report Data, Behavioral, and ERP Results in Music Trainees

#### 3.5.1. Spearman’s Correlation

Given the non-normal distribution and small sample size (n = 19), we examined the Spearman’s rank-order correlations between self-reported and behavioral variables and event-related potentials (ERPs) for music trainees to investigate the relationships between behavioral variables and neural markers of food-related responses (see Figure S2). As shown in Figure S2, RT was positively correlated with the aspects of Having Intentions and Plans to Consume Food and Anticipation of Relief From Negative States and Feelings as a Result of Eating following high-caloric non-targets, high-caloric targets, and low-caloric non-targets (*p* < 0.05). This suggests that heightened anticipation of eating was associated with task performance regardless of the valence (positive or negative) of the anticipated outcomes. Correspondingly, RT following high-caloric targets, low-caloric targets, and low-caloric non-targets was positively correlated with the Guilt From Cravings and/or for Giving Into Them aspect (*p* ≤ 0.01), indicating that guilt stemming from food cravings may serve as a robust indicator of task performance. Additionally, a significant positive correlation was observed between the Anticipation of Positive Reinforcement That May Result From Eating aspect and N2 amplitudes across all four conditions (*p* < 0.01). The aspects of Craving as a Physiological State, Emotions That May Be Experienced Before or During Food Cravings or Eating, and Guilt From Cravings and/or for Giving Into Them were also correlated with N2 amplitudes under certain conditions to varying extents (*p* < 0.05). Furthermore, Body Mass Index (BMI) was negatively correlated with N2 amplitudes following high-caloric non-targets (*ρ* = −0.477, *p* = 0.039) and low-caloric non-targets (*ρ* = −0.477, *p* = 0.039).

#### 3.5.2. Moderation Analysis

Moderation analysis was conducted by fitting Model 1 using the PROCESS macro (version 4.1) to examine whether music learning duration can influence the predictive effect of Guilt From Cravings and/or for Giving Into Them on task performance (RT), as shown in Table 4. Guilt From Cravings and/or for Giving Into Them significantly and positively predicted RT-LT (*β* = 0.590, *p* = 0.010) and RT-HT (*β* = 0.541, *p* = 0.010). However, the positive predictive effect of Guilt From Cravings and/or for Giving Into Them on RT-LN was not significant (*β* = 0.421, *p* = 0.022). Additionally, the interaction term between Guilt From Cravings and/or for Giving Into Them and Music Learning Duration was significant for RT following low-caloric target, high-caloric target, and low-caloric non-target (*p* ≤ 0.05). This indicates that Music Learning Duration can exert an indirect influence on the predictive effect of guilt stemming from food craving on RT.

**Figure 4:**
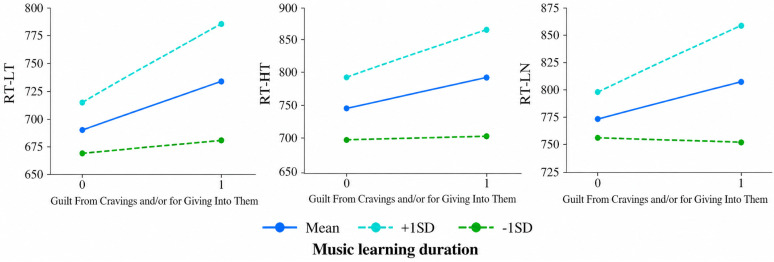
Simple effect analysis. When music learning duration is long, guilt significantly positively predicts RT under all conditions (*p* < 0.01); however, when music learning duration is short, this predictive relationship is no longer significant. The results indicate that only when music trainees have a sufficiently long duration of music learning can their guilt from food cravings positively predict their RT in the 2-back task. Abbreviations: RT, reaction times; LT, low caloric target; HT, high caloric target; LN, low caloric target.

Similarly, to further elucidate the nature of the interaction between Guilt From Cravings and/or for Giving Into Them and music learning duration, we categorized music learning duration into high and low groups based on the mean ± one standard deviation. We then conducted simple slope tests and plotted the simple effects analysis graph. As shown in Figure 4, when music learning duration is long, guilt significantly positively predicts RT under all conditions (*p* < 0.01); however, when music learning duration is short, this predictive relationship is no longer significant. The results indicate that only when music trainees have a sufficiently long duration of music learning can their guilt from food cravings positively predict their RT in the 2-back task.

## 4. Discussion

This study explored the impact of musical training and food craving on WM using a food-cue 2-back task. Musically trained participants exhibited longer RTs. EEG results revealed larger N2 amplitudes in music trainees, indicating greater cognitive conflict. Mediation and moderation analyses showed that food-craving-related intentions and guilt mediated the relationship between musical training and task performance. Together, these findings suggest that musical training amplifies the cognitive interference from food cues, leading to impaired WM performance.

### 4.1. Behavioral Performances

This study provides novel insights into the complex interaction between musical training and food craving, and their combined effects on WM. Contrary to the traditional view that musical training universally enhances WM, our findings indicate that musically trained individuals exhibited longer RTs compared to non-musical participants. This suggests that, rather than simply enhancing cognitive resources, musical training may lead to the allocation of additional cognitive resources towards processing emotionally salient food cues, thus impairing WM efficiency.

Previous studies have demonstrated that long-term musical training is significantly associated with improved performance on n-back tasks, particularly tasks requiring pitch discrimination (Ding et al., 2018). Musicians have also been shown to perform better in WM tasks that require visuomotor coordination, spatial memory, and visual processing speed. In contrast, food craving has consistently been shown to impair WM. For example, chocolate craving selectively impairs cognitive performance related to the visuospatial sketchpad (Tiggemann et al., 2010), and high-calorie food cues impair WM performance in individuals with high food craving tendencies (Meule et al., 2012). While musical training is generally believed to enhance cognitive performance, the extent of these benefits in adults, particularly in tasks involving visual working memory, remains controversial.

Our findings indicate that musically trained participants exhibited prolonged RTs across conditions, suggesting that music trainees required more time to process food-related stimuli. This observation contradicts the conventional view that musical training enhances WM (Gagnon & Nicoladis, 2021). However, it supports the domain-specificity hypothesis, which proposes that the effects of musical training on WM may depend on the type of stimuli and task demands. Specifically, there was a trend for the interference effect to be more pronounced under high-calorie food cues (see Table S1), in line with the findings of Meule et al. (2012), which show that high-calorie foods are more likely to deplete resources in the visuospatial sketchpad. We hypothesize that musical training enhances sensitivity to food cues, thereby exacerbating resource competition between food-related processing and WM tasks.

### 4.2. Neurological Mechanisms

Musically trained participants showed larger N2 amplitudes in response to high-calorie food cues, while no such effect was observed in the non-musical group. The N2 component has been associated with inhibitory control processes (Carbine et al., 2018a), and increased amplitude typically reflects heightened allocation of cognitive resources to inhibit automatic responses to food-related stimuli, particularly when individuals need to suppress these automatic reactions (Carbine et al., 2018a). This aligns with evidence suggesting that musical training enhances prefrontal executive control while simultaneously increasing limbic sensitivity to reward-related stimuli, such as high-calorie food cues (Quinci et al., 2022).

The larger N2 amplitude in the music group may reflect greater cognitive resource allocation required to resolve conflicting information when exposed to food cues. This finding suggests that musically trained individuals may be more susceptible to interference from food cues during WM tasks, requiring more attentional effort to inhibit food-related distractors. Additionally, while non-music trainees exhibited significantly larger beta-wave amplitudes during target trials compared to non-target trials, this difference was absent in the music group. This suggests that musically trained individuals may have a diminished ability to allocate attention efficiently, particularly during tasks that require visual processing (X. Wang et al., 2015). The greater beta amplitude observed in the non-music group may indicate better attentional concentration during target trials (Lundqvist et al., 2018).

This differential pattern of beta activity further supports the idea that musical training may increase neural resource allocation to emotionally salient stimuli, such as food cues. These findings are consistent with Baddeley’s multicomponent model of WM, which posits that when the visuospatial sketchpad is occupied by food-related cues, resources available for WM tasks are reduced, leading to performance decrements (A. Baddeley, 1992). Furthermore, the theta and alpha bands showed no significant differences between music and non-music trainees, suggesting these frequencies did not play a substantial role in the observed effects on WM performance in this study.

This finding is consistent with Tiffany’s cue-reactivity theory (Tiffany, 1990) and extends this theoretical model to the context of food craving. In the experiment, there was a trend for participants in the music training group to show longer RTs in the 2-back task than those in the non-music group. This may be attributed to the heightened food craving in the music training group, which enhances the automatic response to food-related stimuli. Consequently, these stimuli more readily trigger nonautomatic cognitive processes associated with food craving. These processes consume substantial cognitive resources, thereby impairing task performance. Event-related potential results revealed that the music training group exhibited larger N2 amplitudes when processing non-target food-related stimuli, indicating greater cognitive conflict in the face of such distractors, which was consistent with previous research (Folstein & Van Petten, 2008). The results of the P2, P3, and P5 components indicate that these ERP components are primarily associated with cognitive processes related to the task. Specifically, the P3 component exhibited significantly larger amplitudes in target trials, while the P5 component was influenced by the type of food stimuli, with high-calorie food cues eliciting larger P5 amplitudes. These findings further support the influence of musical training and food cues on the allocation of cognitive resources in WM tasks (Ghin et al., 2022). Furthermore, mediation analysis indicated that high food craving in the music training group indirectly influenced task performance through a mediating effect, further supporting the critical role of craving-related nonautomatic processes in cognitive resource allocation. Specifically, music training may enhance the automatic response to food-related stimuli, indirectly leading to the reallocation of cognitive resources and subsequent declines in task performance.

The findings are also consistent with the theoretical framework proposed by Green et al. (Green et al., 2000). Notably, high-calorie food cues elicited a stronger interference effect on WM compared to low-calorie cues, which is in line with the results of Liu et al. (2022). The heightened interference may be attributed to the fact that high-calorie foods are more likely to activate the brain’s reward system, thereby requiring additional cognitive resources for inhibition (Liu et al., 2022).

### 4.3. Executive-Reward-Emotion Triangulation Model

Building upon the extant literature and integrating the current empirical findings, we propose the Executive-Reward-Emotion Triangulation Model to reconcile the complex interplay between musical training, food craving, and working memory (WM), with particular relevance to visual working memory (VWM). This model posits that behavioral performance emerges from the dynamic interaction of three core neurocognitive systems: the executive control network, the reward processing system, and the emotional salience network. Long-term musical training induces experience-dependent neuroplastic adaptations that enhance the functional efficacy of prefrontal-parietal executive circuits (Chen et al., 2017; Rodriguez-Gomez & Talero-Gutierrez, 2022), while concurrently potentiating the responsiveness of limbic and paralimbic regions—such as the amygdala, insula, and orbitofrontal cortex—to emotionally salient and reward-laden stimuli, including high-calorie food cues (Carbine et al., 2018b; Ray & Roche, 2018). This heightened neural sensitivity subjectively manifests as elevated food craving and engages in competitive resource allocation with ongoing VWM tasks, thereby imposing additional cognitive load (Morris et al., 2020).

Consequently, we hypothesize that the performance deficit observed in musically trained individuals—reflected in prolonged RTs—stems not from an executive function deficiency per se, but rather from a “paradox of expertise”: their sharpened emotional and reward perception becomes a “susceptibility burden” in high-conflict contexts, necessitating greater mobilization of cognitive resources for inhibitory control and conflict resolution. This is neurophysiologically evidenced by enlarged N2 amplitudes (Carbine et al., 2018a, 2018b), indicative of enhanced cognitive conflict and compensatory control.

The present results provide compelling support for this model. Contrary to the conventional view that musical training unilaterally enhances cognitive performance (Benz et al., 2016; Gade & Schlemmer, 2021; Schellenberg & Lima, 2024), our data reveal that musically trained participants exhibited significantly longer RTs across all conditions, particularly following non-target trials. This pattern challenges the domain-general transfer hypothesis and underscores the context-dependent nature of musical expertise. Electrophysiological data further elucidate the underlying mechanisms: the enhanced N2 amplitude elicited by high-calorie food cues in the music group signifies intensified cognitive conflict, consistent with the model’s proposition that musically trained individuals deploy deeper emotional and reward-based processing of food stimuli, thereby engendering greater competition for attentional resources and necessitating augmented top-down control from the executive network.

Mediation analyses substantiate this pathway by delineating a clear causal chain: musical training exerts an indirect effect on behavioral performance (prolonged RT) through the mediator of Intentions and Plans to Consume Food—a core dimension of food craving. This pathway elucidates how expertise-induced reward sensitivity (e.g., heightened incentive salience attribution to food) precipitates resource competition (Pasqualitto et al., 2023; Short & Dingle, 2016), ultimately impairing efficiency. Furthermore, moderation analysis revealed that the predictive power of guilt on task performance was significant only among individuals with extended training duration. This dose-dependent effect aligns with the principles of experience-dependent plasticity (Habibi et al., 2018), suggesting that the sharpening of emotion-reward systems is a gradual process that stabilizes with sustained training.

Collectively, these findings depict that musical training shapes a “high-conflict brain”—endowed with a more powerful control engine (executive network) but also a more sensitive accelerator (reward-emotion system). Under neutral conditions, this system excels; however, in environments rich with salient distractors (e.g., high-calorie food cues), the hyper-responsive accelerator demands constant compensatory braking from the control system, metabolizing more cognitive resources and behaviorally manifesting as response delays (Sweller, 2011).

The Executive-Reward-Emotion Triangulation Model offers a nuanced, integrative framework for understanding individual differences in cognition. It reconciles seemingly contradictory findings—that musical training can both enhance and impair performance—within a unified paradigm by emphasizing the contextual equilibrium between executive control and reward-emotion networks. The theoretical contribution of this work lies in revealing the dualistic neurocognitive impact of musical expertise: it is not merely a training regimen for cognitive control but also a potent modulator of affective and incentive processing (Neves et al., 2025). Future research should employ multimodal neuroimaging (e.g., fMRI) to directly quantify the dynamic interactions between these large-scale networks and identify additional moderating variables (e.g., personality traits, genetic polymorphisms) that govern this delicate balance, thereby advancing a more comprehensive understanding of the architecture of human cognition and emotion.

### 4.4. Limitations

This study has several limitations that should be addressed in future research. First, the cross-sectional design limits causal inference. Future studies should adopt longitudinal or experimental designs to clarify the directionality of the relationships among musical training, food craving, and working memory performance, and to determine whether musical experience modulates the impact of food-cue interference over time. Second, the relatively small sample size (N = 38) and the exclusive focus on university students may limit the generalizability of the present findings. Future research should recruit larger and more diverse samples across different age groups, educational backgrounds, and levels of eating-related vulnerability to examine the robustness and broader applicability of these effects. Third, music-related individual differences were indexed only by a broad measure of musical training history. Future studies should assess musical experience in a more refined manner by considering the type of training (e.g., instrumental vs. vocal), duration, intensity, age of onset, and, importantly, musical sophistication, so as to better distinguish formally untrained individuals who may nevertheless possess relatively high levels of music-related skills or engagement. Fourth, the present study relied on a visual 2-back task to assess working memory. Future studies should include a wider range of working memory paradigms, such as verbal, auditory, and updating tasks, to determine whether the observed effects are specific to visually induced food-cue interference or generalize across working memory domains. Fifth, although the present findings provide behavioral and ERP evidence, the underlying mechanisms remain to be further clarified. Future research could integrate multimodal approaches, such as combining EEG with self-report, physiological, or neuroimaging measures, to better characterize how food craving, attentional control, and music-related experience jointly influence working memory processing. Finally, future studies may also explore intervention-oriented questions, for example, whether music-based cognitive training or craving-regulation strategies can reduce food-related interference and improve working memory performance under motivationally salient conditions.

## 5. Conclusions

This study demonstrates that musical training does not universally enhance EFs; instead, it may increase sensitivity to emotionally salient distractors, such as food cravings, particularly in contexts that require self-regulation. These findings highlight the importance of considering individual differences and contextual factors when assessing the cognitive outcomes of musical training. Theoretically, our results challenge the assumption of a domain-general transfer effect from musical training to WM, suggesting that these effects are influenced by both task demands and internal states, such as craving intensity. From a practical perspective, the findings suggest that interventions aimed at individuals with musical training should include strategies to manage food-related cravings, particularly in environments rich with high-calorie food cues, which may impair WM performance. Future research should explore integrated interventions that address both WM enhancement and craving regulation, ultimately improving cognitive control and task performance in conditions involving motivational conflict.

## Figures and Tables

**Table 1:** Descriptive statistics of democratic information and self-report data.

		Music Trainees (n = 19)	Non-Music Trainees (n =19)	*t*-Test
		M (SD)	M (SD)	
Age		19.58 (1.465)	19.74 (1.851)	−0.292
Gender		1.42 (0.507)	1.47 (0.516)	0.959
BMI		21.74 (1.98)	22.03 (2.34)	−0.417
Music Learning Duration		6.95 (4.37)	0.00 (0.00)	6.183 ***
FCQ-T	Intentions & Plans	9.95 (1.58)	7.37 (1.85)	5.031 ***
	Anticipation of Positive Reinforcement	17.37 (2.72)	17.63 (3.04)	0.505
	Anticipation of Relief From Negative States and Feelings	10.42 (1.76)	10.47 (2.03)	0.277
	Lack of Control	14.21 (3.52)	17.21 (3.71)	−3.115 **
	Thoughts or Preoccupation	16.79 (6.06)	16.21 (5.71)	0.74
	Physiological State	13.26 (2.72)	12.42 (2.08)	1.063
	Emotions	12.32 (2.46)	12.53 (2.97)	−0.178
	Cues	59.53 (16.72)	58.79 (16.76)	−0.485
	Guilt	8.58 (2.42)	5.21 (1.61)	6.486 ***
Hunger		5.32 (1.974)	4.47 (2.17)	1.252

Notes: ** *p* < 0.01; *** *p* < 0.001. Nine aspects of FCQ-T are Having Intentions and Plans to Consume Food, Anticipation of Positive Reinforcement That May Result From Eating, Anticipation of Relief From Negative States and Feelings as a Result of Eating, Lack of Control Over Eating, Thoughts or Preoccupation With Food, Craving as a Physiological State, Emotions That May Be Experienced Before or During Food Cravings or Eating, Cues That May Trigger Food Cravings, Guilt From Cravings and/or for Giving Into Them, respectively.

**Table 2:** Descriptive statistics (M ± SD) of N2, P2, P3, and P5 amplitude for all participants in the 2-back task.

ERP Component	Stimuli	Type	Group	
			Music Trainees	Non-Music Trainees
N2	High-calorie food	Target	0.49 ± 7.52	−1.23 ± 7.45
		Non-target	−1.95 ± 8.67	−1.86 ± 8.83
	Low-calorie food	Target	1.09 ± 7.25	−1.26 ± 8.55
		Non-target	−1.39 ± 8.31	−1.74 ± 8.58
P2	High-calorie food	Target	2.18 ± 6.61	1.37 ± 4.38
		Non-target	0.56 ± 6.16	0.42 ± 5.05
	Low-calorie food	Target	1.50 ± 6.17	0.53 ± 5.48
		Non-target	−0.44 ± 5.40	−0.26 ± 5.42
P3	High-calorie food	Target	4.15 ± 8.18	2.71 ± 6.81
		Non-target	0.58 ± 7.75	−0.54 ± 7.76
	Low-calorie food	Target	3.99 ± 7.49	1.05 ± 6.71
		Non-target	−0.05 ± 8.24	−1.10 ± 8.82
P5	High-calorie food	Target	5.31 ± 7.95	4.74 ± 5.73
		Non-target	1.84 ± 7.09	2.63 ± 7.26
	Low-calorie food	Target	3.65 ± 8.19	3.17 ± 6.73
		Non-target	−1.04 ± 7.88	1.51 ± 7.63

**Table 3:** Descriptive statistics(M ± SD) of beta amplitudes on Oz and theta amplitudes on Cz for all participants in the 2-back task.

Band	Stimuli	Type	Group	
			Music Trainees	Non-Music Trainees
Oz	High-calorie food	Target	−0.240 (0.606)	−0.058 (0.491)
		Non-target	−0.233 (0.531)	−0.051 (0.452)
	Low-calorie food	Target	−0.272 (0.558)	−0.356 (0.556)
		Non-target	−0.263 (0.341)	−0.265 (0.401)
Cz	High-calorie food	Target	0.433 (0.578)	0.408 (0.668)
		Non-target	0.378 (0.315)	0.120 (0.658)
	Low-calorie food	Target	0.567 (0.754)	0.338 (0.455)
		Non-target	0.428 (0.448)	0.244 (0.505)

**Table 4:** The Moderating Role of Musical Training Duration in the Relationship between Food Craving and Behavioral Performance.

	RT-LT		RT-HT		RT-LN	
	*β*	*t*	*β*	*t*	*β*	*t*
Guilt From Cravings and/or for Giving Into Them	0.590	3.365 **	0.541	2.847 *	0.421	1.999
Music Learning Duration	0.125	0.685	0.213	1.079	0.098	0.450
Guilt From Cravings and/or for Giving Into Them × Music Learning Duration	0.494	2.963 ***	0.531	2.936 **	0.511	2.548 *
*R* ^2^		0.639				0.479
*R*^2^ Adjusted		0.566				0.374
*F*		8.837 **		6.761 **		4.591 *

Note: * *p* < 0.05; ** *p* < 0.01; *** *p* < 0.001.

## Data Availability

The data will be made available on request.

## Supplementary Materials

The following supporting information can be downloaded at: https://www.mdpi.com/article/10.3390/bs16050659/s1. Figure S1. Trial structure of the 2-back task. Figure S2. Spearman’s correlation between all the variables. Table S1. Descriptive statistics (M ± SD) of RT for all participants in the 2-back task. Table S2. Descriptive statistics (M ± SD) of ACC for all participants in the 2-back task.
