# Supplementary material for: Musical Training Amplifies Food Cue-Related Interference in Working Memory
Source: Behav Sci (Basel). 2026 Apr 27;16(5):659. doi: 10.3390/bs16050659 (PMC13203654; doi:10.3390/bs16050659)
Supplement: Supplementary file 1 [file behavsci-16-00659-s001.zip › behavsci-4231476-supplementary.pdf]

Musical training amplifies food cue–related interference in working memory

Supplementary Materials

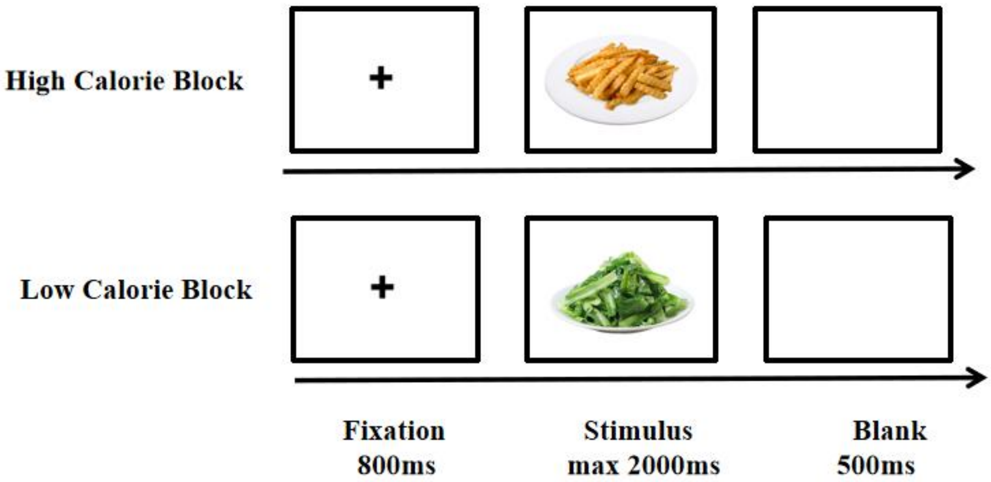

Fig. S1 Trial structure of the 2-back task.

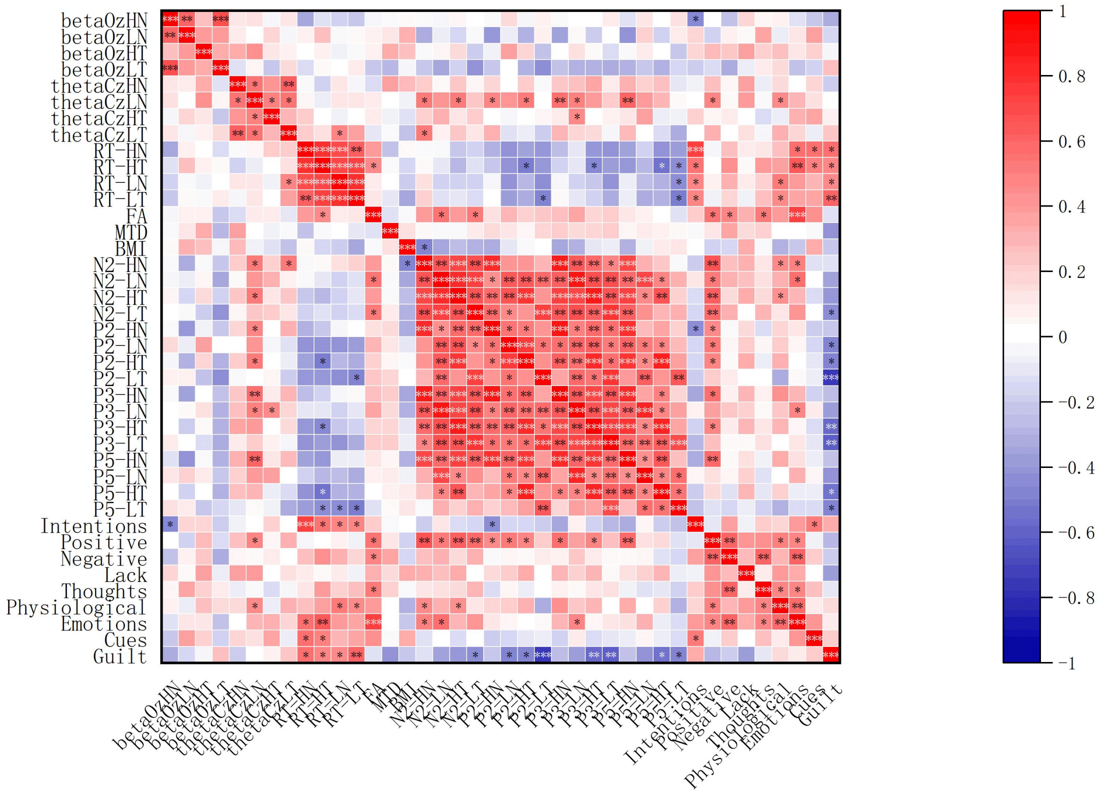

Fig. S2 Spearman's correlation between all the variables.

Abbreviation: HN, high caloric non-target; HT, high caloric target; LN, low caloric target; LT, low caloric target; FA, Food Addiction; MTD, Music Training Duration. *p<0.05; ** p<0.01.

Table S1 Descriptive statistics(M±SD) of RT for all participants in the 2-back task.

Stimuli	Type	Group	
		music trainees	Non-music trainees
High-calorie food	Target	697.79 (203.339)	617.53 (152.020)
	Non-target	756.58 (208.326)	624.32 (136.390)
Low-calorie food	Target	657.95 (167.502)	618.79 (122.902)
	Non-target	736.53 (185.796)	643.42 (120.773)

Table S2 Descriptive statistics(M±SD) of ACC for all participants in the 2-back task.

Stimuli	Type	Group	
		music trainees	Non-music trainees
High-calorie food	Target	0.847 (0.125)	0.850 (0.106)
	Non-target	0.842 (0.100)	0.862 (0.051)
Low-calorie food	Target	0.822 (0.150)	0.826 (0.129)
	Non-target	0.847 (0.110)	0.877 (0.064)
